# Deep sequencing of circulating tumour DNA as a biomarker of clinical outcome to transarterial chemoembolisation in hepatocellular carcinoma

**DOI:** 10.1038/s41698-025-00961-2

**Published:** 2025-07-01

**Authors:** Rohini Sharma, Sultan N. Alharbi, Ksenia Ellum, Leila Motedayen-Aval, Andrea Casadei-Gardini, David J. Pinato, Dominik Bettinger, Bertram Bengsch, Rishi Patel, Joanne Evans

**Affiliations:** 1 Department of Surgery and Cancer, Imperial College London, London, UK; 2 Department of Oncology, IRCCS San Raffaele Scientific Institute Hospital, Vita-Salute San Raffaele University, Milan, Italy; 3 Department of Translational Medicine, Universita’ degli Studi del Piemonte Orientale “A. Avogadro”, Novara, Italy; 4 Faculty of Medicine, Clinic for Internal Medicine II, Gastroenterology, Hepatology, Endocrinology, and Infectious Disease, University Medical Centre Freiburg, Freiburg, Germany; 5 German Cancer Consortium (DKTK), Heidelberg, Germany; 6 Eastman Dental School, University College London, London, UK

**Keywords:** Prognostic markers, Hepatocellular carcinoma

## Abstract

Survival following transarterial chemoembolisation (TACE) for hepatocellular cancer (HCC) is variable. We explored targeted sequencing of circulating tumour DNA (ctDNA) as a prognostic biomarker. Plasma samples (*n* = 97) were collected at baseline and following TACE. Targeted, ultra-deep sequencing was conducted on 18 somatic mutations related to the molecular pathogenesis of HCC. Median progression-free survival and overall survival were 11.6 months (95% CI: 5.83–21.2) and 34 months (95% CI: 22.35–45.60), respectively. CTNNB1 and ARID1A were the most frequently mutated genes, present in 25% of baseline circulating samples, followed by SF3B1 (20%) and TERT (18%). The presence of mutations in CTNNB1, TP53 ARID1A, and KEAP1 predicted for poor OS on univariable analysis. Findings suggest that ctDNA profiling of known genetic drivers of HCC may serve as a valuable prognostic biomarker prior to TACE and may assist with the stratification of patients following further evaluation in larger studies.

## Introduction

Transarterial chemoembolisation (TACE) is the recommended first-line treatment for intermediate stage HCC^1^. TACE involves the infusion of small embolic particles or liquid embolics into the hepatic artery with or without chemotherapy with the intent of prolonging survival through local cancer control^2^. Patients may undergo several cycles of TACE every 1–3 months, depending on tumour response, and as such, TACE is one of the most widely used treatments for HCC. However, there is marked variability in the prognosis of patients with intermediate stage disease, ranging from 11 to 45 months^1^. A number of prognostic scores have been explored with a view to stratifying those patients most likely to benefit from TACE and those that would benefit from upfront systemic therapy^3–8^. This is of particular relevance in the advent of combination immunotherapy, which offers a median overall survival of 19.2 months in patients with preserved liver function^9^. However, the performance of these prognostic models is variable, and no one test has undergone external validation. There is a need for an alternative, early evaluation strategy based on tumour biology, which can prevent ineffective treatment and facilitate clinical decision-making.

The genetic landscape of HCC is well described, with mutations in the catenin beta 1 (CTNNB1), tumour protein p53 (TP53), and telomerase reverse transcriptase (TERT) being the most commonly observed driver mutations in HCC^10–12^. However, most studies investigating the impact of genetic alterations utilise tumour tissue, a significant clinical limitation in HCC as most diagnoses are based on radiological criteria, with only a minority of patients undergoing liver biopsy for histopathological diagnosis^13^. Moreover, the use of tumour tissue is inherently limited by intratumoural heterogeneity and the invasive nature of the procedure, impacting on the use of tissue for longitudinal assessment of genetic alterations.

Circulating cell-free DNA (cfDNA) is released from both healthy and malignant cells through apoptosis and necrosis into the blood stream and is gaining traction as a non-invasive marker of tumour biology, obviating the need for repeated tumour biopsies^14^. Absolute levels of cfDNA can be used to distinguish malignancy from benign inflammatory diseases and guide treatment response. Tumour-derived cfDNA (ctDNA) can be distinguished from cfDNA by identification of somatic alterations present in the tumour of interest, but not in matched somatic tissue^14^. ctDNA can easily be collected in the clinic to monitor both treatment response and tumour recurrence and has been shown promise in a number of malignancies, including HCC^15^. There has only been a single study investigating the utility of ctDNA for predicting outcome to TACE^16^. However, ctDNA was defined only by the presence of two-point mutations in *TERT*. The aim of this study was to determine whether the detection of targeted mutations in known driver genes in ctDNA is predictive of clinical outcomes from TACE. The results of this study may provide an alternate model for predicting TACE response based on tumour biology.

## Results

cfDNA was detected at baseline in 97 patients who were included in the final analysis. Mean age was 69 years (range 45–86). Majority had cirrhosis (83%) with preserved liver function (Child-Pugh A, 63%) secondary to hepatitis C (34%). Most patients underwent TACE first line (98%) for BCLC-B disease (64%). When considering survival outcomes, median PFS was 13.2 months (95% CI: 9.9–16.3) and median OS was 38.5 months (95% CI: 16.3–60.9)(Table 1).

**Table 1:** Distribution of demographic and clinical variables among enroled HCC patients (*n* = 97)

	Number (%)
Country:	
United Kingdom	54 (56)
Italy	27 (29)
Germany	16 (17)
Gender:	
Male	73 (75)
Female	24 (25)
Median age (years, range)	69 (45–86)
Cirrhosis	80 (83)
Aetiology:	
HBV	12 (12)
HCV	33 (34)
Alcohol	18 (19)
NASH	17 (18)
Other	13 (13)
BCLC stage:	
A	14 (14)
B	62 (64)
C	14 (14)
Child-Pugh class:	
A	61 (63)
B	33 (34)
Number of nodules	
≤2	60 (62)
>2	9 (9)
Median tumour size (cm, range)	4 (1.2–16)
AFP, median (ng/mL) (range)	9.4 (1–10809)

*HBV* Hepatitis B virus, *HCV* Hepatitis C virus, *NASH* Non-alcoholic steatohepatitis, *AFP* alpha-fetoprotein, *BCLC* Barcelona Clinic Liver Cancer score.

### Relationship between cfDNA and clinical outcomes following TACE

**Fig. 1:**
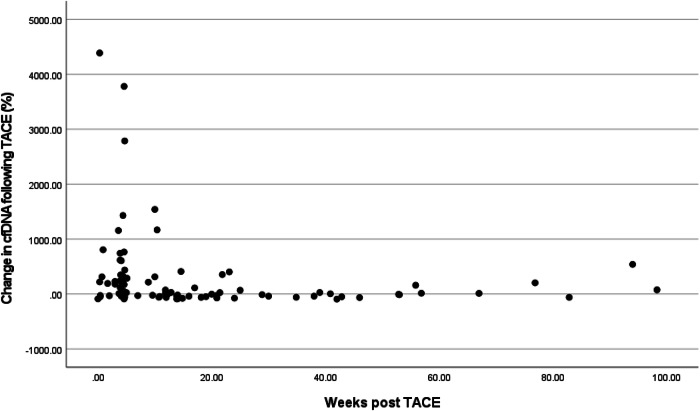
Change in cfDNA (%) was greatest in the early weeks following TACE (weeks).

We considered the dynamic change in cfDNA levels following TACE. Samples were taken over a range of time intervals following TACE, median 11 weeks (range 3 days to 98 weeks). The baseline median cfDNA level was 581 ng/L (IQR 279–970 ng/L). We documented an increase in cfDNA release post TACE, which reduced with increasing time from the procedure (Fig. 1). In terms of treatment response, complete response was observed in 41% of patients, partial response in 25%, and stable disease in 9%; 22% had progressive disease. No association was seen between baseline cfDNA levels or changes in cfDNA levels over time with response. Similarly, no relationship between cfDNA levels and survival outcomes was observed.

### Baseline mutational landscape predicts for clinical outcome to TACE

Of the 97 patients evaluable for efficacy, 23 (24%) patients had samples of adequate quality at T0, 22 (23%) at T1, and 6 (6%) at T2 for ctDNA (Table 2). At T0, the VAF was 60.8%, at T1 it was 44.7% and T2, 13.2%.

**Table 2:** Distribution of demographic and clinical variables from a subset of patients who underwent targeted sequencing (*n* = 23)

	Number (%)
Gender:	
Male	17 (74)
Median age (years, range)	67 (50–86)
Cirrhosis	21 (91)
Aetiology:	
HBV	1 (4)
HCV	9 (39)
Alcohol	5 (22)
NASH	7 (30)
Other	1 (4)
BCLC stage:	
A	5 (22)
B	15 (65)
C	1 (4)
Child-Pugh class:	
A	22 (96)
B	1 (4)
Median tumour size (cm, range)	3.5 (1.2–14)
AFP, median (ng/mL) (range)	7.5 (1.9–803)

*HBV* Hepatitis B virus, *HCV* Hepatitis C virus, *NASH* Non-alcoholic steatohepatitis, *AFP* alpha-fetoprotein, *BCLC* Barcelona Clinic Liver Cancer score.

When considering baseline factors, low VAF at baseline was significantly associated with a significantly larger tumour size (*p* = 0.03). A significant difference was observed between increasing VAF and raised AFP (Z = -4.1, *p* < 0.001). Of significance, high VAF at T0 was associated with worse OS (HR 3.1, 95%CI: 1.0–9.7, *p* = 0.048) but not or PFS or response to TACE.

### Impact of TACE on the circulating mutational landscape

**Fig. 2:**
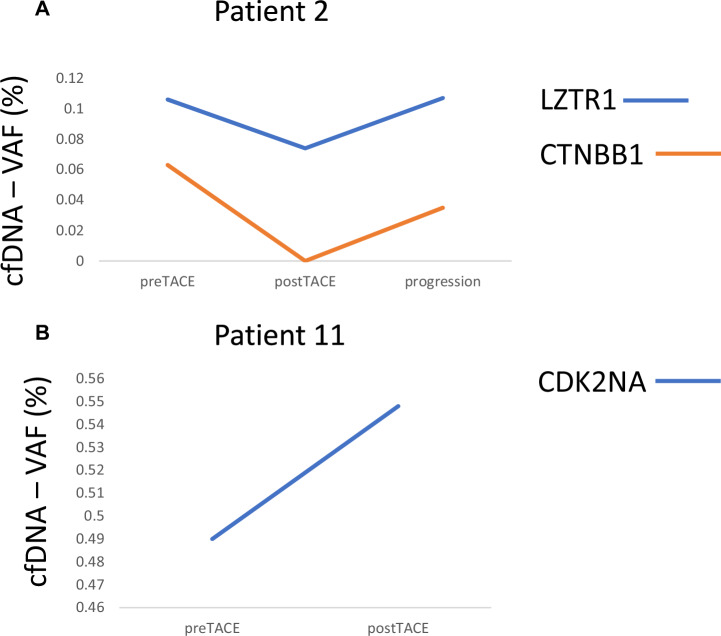
The Variant Allele Frequency (VAF) of specific mutations changes during the course of the treatment. For instance, the VAF of LZTR1 and CTNBB1 reduces immediately following TACE, then increases at treatment failure in patients 2 (long PFS) (**A**). Whilst in patient 11, who did not respond to TACE VAF of CDK2NA increased (**B**).

We then investigated the presence of known putative mutations for HCC in pre-TACE samples that were available for 23 patients. At baseline, a total of 54 mutations in the targeted genes were identified. The set of individual mutation statuses for each patient is shown in Table S1. Of the targeted genes of interest, the most genes with the most frequent actionable mutations included ARID1A (14.8%), followed by TERT (9.2%), SF3B1 (9.2%), and ATM (9.2%) (Table 3). After filtering, temporal changes in VAF of specific variants were observed (Fig. 2A and B).

**Table 3:** Frequency of putative genetic variants identified in plasma ctDNA from patients with HCC (*n* = 23)

Gene	Position	DNA change	Variant Classification	Protein change	Number (%) of HCC patients with mutation (*n* = 22)	Number (%) HCC patients with gene affected (*n* = 22)
APC	chr5:112775613	A > T	Intron	-	2 (9%)	3 (14%)
	chr5:112828978	T > A	Intron	-	1 (5%)	
	chr5:112841202	G > A	Missense	p.D1870N	1 (5%)	
	chr5:112843530	C > T	Nonsense	p.Q2646*	1 (5%)	
ARID1A	chr1:26696303	T > G	5’UTR	-	2 (9%)	8 (36%)
	chr1:26696243	delG	5’UTR	-	1 (5%)	
	chr1:26696939	C > T	Missense	p.P179L	1 (5%)	
	chr1:26781181	delA	3’UTR	-	1 (5%)	
	chr1:26781181	insA	3’UTR	-	1 (5%)	
	chr1:26781180	CA > AA	3’UTR	-	1 (5%)	
	chr1:26775548	T > A	Intron	-	1 (5%)	
	chr1:26765502	T > A	Intron	-	1 (5%)	
	chr1:26780431	A > G	Missense	p.D1961G	1 (5%)	
	chr1:26764279	delTTA	Intron	-	1 (5%)	
	chr1:26775642	G > T	Missense	p.A1470S	1 (5%)	
	chr1:26766179	A > T	Intron	-	1 (5%)	
	chr1:26771867	insGA	Intron	-	1 (5%)	
	chr1:26781594	G > A	3’UTR	-	1 (5%)	
ARID2	chr12:45907298	G > A	3’UTR	-	1 (5%)	5 (23%)
	chr12:45776208	G > C	Intron	-	1 (5%)	
	chr12:45850291	T > C	Missense	p.I723T	1 (5%)	
	chr12:45851855	C > T	Silent	p.C1244C	1 (5%)	
	chr12:45893906	delAA	Intron	-	1 (5%)	
ATM	chr11:108368056	T > C	3’UTR	-	1 (5%)	11 (50%)
	chr11:108366325	T > A	3’UTR	-	1 (5%)	
	chr11:108226129	delCT	Intron	-	1 (5%)	
	chr11:108368079	G > A	3’UTR	-	1 (5%)	
	chr11:108317331	T > C	Intron	-	2 (9%)	
	chr11:108229848	delTTT	Intron	-	2 (9%)	
	chr11:108353824	C > G	Silent	p.L2910L	1 (5%)	
	chr11:108226465	A > G	Intron	-	1 (5%)	
	chr11:108236535	delAA	Intron	-	1 (5%)	
	chr11:108253979	A > G	Silent	p.E688E	1 (5%)	
	chr11:108336314	G > A	Intron	-	1 (5%)	
AXIN1	chr16:292909	delTC	Intron	-	1 (5%)	5 (23%)
	chr16:287828	delG	3’UTR	-	1 (5%)	
	chr16:287827	TG > GG	3’UTR	-	1 (5%)	
	chr16:291664	G > A	Intron	-	1 (5%)	
	chr16:291690	G > A	Intron	-	1 (5%)	
	chr16:292771	C > T	Intron	-	1 (5%)	
	chr16:346333	C > T	Silent	p.G231G	1 (5%)	
	chr16:290341	delC	Intron	-	1 (5%)	
	chr16:289748	CA > AG	Intron	-	1 (5%)	
CDKN2A	chr9:21971112	G > A	Missense	p.H32Y	1 (5%)	4 (18%)
	chr9:21995246	C > G	5’UTR	-	2 (9%)	
	chr9:21969809	delG	Intron	-	1 (5%)	
	chr9:21994124	G > A	Intron	-	1 (5%)	
	chr9:21994137	A > G	Splice Site	-	1 (5%)	
CTNNB1	chr3:41222081	delA	5’UTR	-	1 (5%)	5 (23%)
	chr3:41222080	TA > AA	5’UTR	-	1 (5%)	
	chr3:41222080	insA	5’UTR	-	1 (5%)	
	chr3:41233410	C > A	Missense	p.T384N	1 (5%)	
	chr3:41237312	T > C	Intron	-	1 (5%)	
	chr3:41195090	G > C	5’UTR	-	1 (5%)	
	chr3:41221852	T > A	5’UTR	-	1 (5%)	
HNF1A	chr12:120994312	delG	Frame Shift	p.P291fs	1 (5%)	2 (9%)
	chr12:120993492	delC	Intron	-	1 (5%)	
	chr12:120997748	insCC	Intron	-	1 (5%)	
JAK1	chr1:64869304	G > A	Intron	-	1 (5%)	3 (14%)
	chr1:64834085	G > A	3’UTR	-	1 (5%)	
	chr1:64875319	G > A	Intron	-	1 (5%)	
	chr1:64860150	delG	Frame Shift	p.P430fs	1 (5%)	
KEAP1	chr19:10500119	G > A	Intron	-	1 (5%)	4 (18%)
	chr19:10503405	C > T	5’Flank	-	1 (5%)	
	chr19:10503181	C > T	Intron	-	1 (5%)	
	chr19:10489604	A > G	Intron	-	1 (5%)	
LZTR1	chr22:20982316	C > A	5’UTR	-	1 (5%)	3 (14%)
	chr22:20992853	C > T	Silent	p.F403F	1 (5%)	
	chr22:20997565	C > G	3’UTR	-	1 (5%)	
	chr22:20997892	T > C	3’UTR	-	1 (5%)	
PDGFRA	chr4:54278426	T > G	Missense	p.N689K	1 (5%)	1 (5%)
PIK3CA	chr3:179204485	CC > AA	Intron	-	1 (5%)	6 (27%)
	chr3:179237557	C > T	3’UTR	-	1 (5%)	
	chr3:179236267	T > C	3’UTR	-	1 (5%)	
	chr3:179195993	A > G	Intron	-	1 (5%)	
	chr3:179197765	A > G	Intron	-	1 (5%)	
	chr3:179235739	C > T	3’UTR	-	1 (5%)	
PTEN	chr10:87967851	G > T	3’UTR	-	1 (5%)	3 (14%)
	chr10:87966104	C > T	3’UTR	-	1 (5%)	
	chr10:87966298	delG	3’UTR	-	1 (5%)	
SF3B1	chr2:197400448	insA	Intron	-	1 (5%)	9 (41%)
	chr2:197402110	T > C	Missense	p.K700E	2 (9%)	
	chr2:197400447	delT	Intron	-	1 (5%)	
	chr2:197405293	T > C	Silent	p.Q473Q	1 (5%)	
	chr2:197390330	T > G	3’UTR	-	1 (5%)	
	chr2:197419393	delCA	Intron	-	1 (5%)	
	chr2:197398107	T > C	Silent	p.E1048E	1 (5%)	
	chr2:197402637	T > C	Missense	p.K666E	2 (9%)	
	chr2:197390456	G > A	3’UTR	-	1 (5%)	
SMARCA4	chr19:11019395	T > C	Intron	-	1 (5%)	5 (23%)
	chr19:11025373	A > C	Intron	-	1 (5%)	
	chr19:10996186	A > G	Intron	-	1 (5%)	
	chr19:10961396	T > G	Intron	-	1 (5%)	
	chr19:11007936	C > A	Missense	p.P679H	1 (5%)	
TERT	chr5:1282922	A > G	Intron	-	1 (5%)	7 (32%)
	chr5:1295113	G > A	5’Flank	-	1 (5%)	
	chr5:1294720	delC	Intron	-	1 (5%)	
	chr5:1255402	T > C	Silent	p.A1014A	1 (5%)	
	chr5:1295234	A > G	5’Flank	-	1 (5%)	
	chr5:1296371	A > G	5’Flank	-	1 (5%)	
	chr5:1294550	delG	Frame Shift	p.E113fs	1 (5%)	
	chr5:1253800	delC	Frame Shift	p.T1110fs	1 (5%)	
TP53	chr17:7675124	T > C	Missense	p.Y163C	1 (5%)	1 (5%)

ins insertion.

del deletion.

*Stop codon.

We then performed a univariable and multivariable Cox regression analysis of OS and PFS using known factors that impact survival outcomes, including aetiology, BCLC stage, serum AFP and tumour size. On univariable analysis, VAF (HR = 3.1, 95%CI: 1.0–9.7, *p* = 0.05), the presence of mutations in CTNNB1 (HR = 5.3, 95%CI: 1.4–20.6, *p* = 0.02), TP53 (HR = 5.3, 95%CI: 1.4–10.10, *p* = 0.03), ARID1A (HR = 5.1, 95%CI: 1.3–21.0, *p* = 0.03) and KEAP1 (HR = 22.0, 95%CI: 2.2–221.0, *p* = 0.004) predicted for poor OS. On multivariable analysis, TP53 (HR = 53.3, 95% CI: 2.0–1396.1, *p* = 0.02), as was the presence of portal vein thrombosis (HR = 26.3, 95% CI: 2.4–292.9, *p* = 0.06), were retained as a negative prognostic factor. However, mutations in TP53 was observed in only one patient and these results require further evaluation (Table 4). Similarly, the presence of TP53 was observed to be associated with worse PFS (HR = 21.5, 95% CI: 1.3–343.7, *p* = 0.03). Neither VAF nor the presence of any other mutation was found to be predictive of PFS (supplementary Table 2).

**Table 4:** Univariable and multivariable Cox regression analysis for overall survival

	Overall survival					
	Univariable analysis			Multivariable analysis		
	HR	95%CI	*p*-value	HR	95%CI	*p*-value
CTNNB1 mutation	**5.3**	1.4–20.6	**0.02**	2.4	0.4–13.6	0.2
TP53 mutation	5.3	1.4–10.1	**0.03**	53.3	2.0–1396.1	**0.02**
KEAP1 mutation	**22.0**	2.2–221.0	**0.004**	2.4	0.4–1285.3	0.8
ARID1A mutation	5.1	1.3–21.0	**0.03**	4.4	0.7–26.7	0.1
VAF	3.1	1.0–9.7	0.05			
BCLC	1.2	0.2–8.3	0.9			
Presence of PVT	13.0	1.8–93.8	**0.02**	26.3	2.4–292.9	**0.006**

Bold values identify statistical significance, *p* < 0.05.

## Discussion

TACE is the most widely used treatment for HCC worldwide. However, there is no consensus as to the optimal schedule or number of rounds of TACE that should be prescribed. Response to treatment is variable, and with the advent of immunotherapy, there is a need for better markers of response with the view to limit locoregional therapies in order to preserve liver function. In this study, we have considered the impact of known driver mutations in circulating ctDNA as predictors for outcome to TACE. There is increasing interest in ctDNA as a prognostic biomarker. However, most studies undertake an untargeted approach, which necessitates confirmation of mutations as pathogenic through the use of matched tumour samples^17^. Using plasma samples from patients with HCC, we undertook a targeted sequencing approach whereby only those mutations known to be driver mutations in HCC development and progression were considered.

We have shown that the cfDNA levels following TACE alter with the time the plasma sample is taken following the TACE, with levels falling to near baseline 30 days following the procedure. This is consistent with the mechanism of action of TACE, which initiates an acute ischaemic event resulting in significant inflammation and apoptosis. In our large cohort, we did not find any association between cfDNA levels and clinical outcome, inconsistent with the literature^16^. This is likely to be attributable to the varying times at which cfDNA samples were taken.

Subsequently, we considered the presence of a mutational panel of genes known to be driver mutations for HCC. We illustrated that high levels of VAF were associated with increased serum AFP levels and worse clinical outcome, consistent with previous studies in HCC^18^. VAF reflects the mutational burden of the tumour. It is established that clinical outcome is associated with more heterogenous tumour carrying multiple mutations, suggesting a more aggressive clinical course^19^. A key limitation of our work was the poor yield of ctDNA, with only 23 patients having material suitable for targeted sequencing. There are a number of factors that may have attributed to low ctDNA yield. Firstly, tumour burden; whilst the majority of cases in our study were BCLC-B, there was a large proportion of patients with BCLC-A disease, which has impacted on ctDNA yield, whereby previous work has been in advanced stage cancers, which have a great amount of circulating tumour DNA^20^. We only collected 1 ml of plasma, which will further reduce the ctDNA amount. Sample collection and handling have also been shown to have a significant impact on the yield and quality of ctDNA^17,21^. Samples were not collected using a standardised protocol across sites, and storage time varied, which will negatively impact on ctDNA yield.

When considering individual mutations, we observed that the presence of CTNNBI, TP53, KEAP1, and ARID1A mutations was predictive of worse OS on univariable analysis, whilst on multivariable analysis TP53 was retained as a negative prognostic marker, as was the presence of portal vein thrombosis (PVT). The frequency of mutations detected was lower than anticipated for example, *TERT* mutations are the most prevalent mutation in HCC, occurring in approximately 60% of cases^22^. However, we only observed mutations in 9%. Similarly, we had anticipated a higher frequency of *CTNNB1* and *TP53* mutations based on the literature^22^. This is likely a reflection of the small number of patients included in the analysis. However, the low prevalence of *TERT* mutations in our cohort may also pertain the high GC content of the region, which makes the region difficult to denature and amplify, impacting on sequencing outcomes. Importantly, we identified the presence of mutations in *TP53* as a negative prognostic factor consistent with previous work illustrating that cancers harbouring this mutation have an exhausted immune phenotype and poor survival outcomes^23^. Further work suggests that *TP53* mutations are characteristic of the macrotrabecular-massive subtype of HCC, a subtype characterised by high AFP levels and aggressive immunologic features^24^. Cells harbouring mutations in TP53 escape apoptosis following DNA damage, resulting in malignancy. Mutant TP53 protein further accumulates within the nucleus, making it a highly specific biomarker of malignant transformation^25^. Given the high prevalence of TP53 mutations in cancer, there has been considerable interest in the development of therapeutics targeting this pathway, none of which have been successfully translated into the clinic, but hopefully will open novel therapeutics for the management of HCC in the future^26^.

The key limitation of our study is the small sample size and lack of a uniform protocol for sample collection, which directly impacted the ctDNA yield obtained. Moving forward, this technology may be of greatest value in the intermediate/advanced stage disease, where there is a clinical need regarding the sequencing of TACE and systemic therapies, and less useful for prognostication in early-stage HCC. However, a strength of this work is the depth of sequencing undertaken for known driver mutations in HCC. Nonetheless this study illustrates the utility of targeted sequencing of ctDNA as a prognostic marker in intermediate-stage HCC. This work lays the foundation for a larger, prospective study.

## Methods

### Patient characteristics

In this prospective, multicentre study, 103 patients (over 18 years of age) with confirmed radiologic diagnosis of HCC^27^ suitable for TACE according to the BCLC treatment algorithm^1^ were recruited from three specialist liver cancer clinics: Imperial College NHS Trust, United Kingdom, *N* = 55, Vita-Salute San Raffaele University, Italy, *N* = 28, and University of Freiberg, Germany *N* = 20, from April 2015 to July 2020. The decision for TACE was decided following multidisciplinary review at each site. All patients received conventional super-selective TACE, consisting in intra-arterial injection of lipiodol plus doxorubicin (60 mg fixed dose) followed by injection of an embolic agent (gelfoam) to arrest blood flow to the tumour.

The response was defined according to mRECIST at the first scan by either CT or MRI, according to physician choice, 8–12 weeks after the last TACE. PFS was defined by the time from the first TACE to the first occurrence of documented disease progression based on mRECIST criteria or death from any cause, whichever occurred first. Median PFS was calculated using the Kaplan-Meier method and was defined as the time from TACE to either progression or death, whichever came first. OS was defined from the time of the TACE visit till death from any cause. PFS was censored at the time of the last tumour assessment in those patients who had not progressed by the time of database lock (3/8/2022), whereas OS was censored at the time of last patient contact. Survival follow-up was carried out every 12 weeks following TACE. Ethical approval was obtained by the Health Research Authorities (Sheffield Research Ethics Committee, reference 198951) and was conducted in accordance with the 1975 Declaration of Helsinki.

### Sample collection

A 10 mL blood sample was collected from all subjects on the day of recruitment in an ethylene diamine tetra-acetic acid tube, and 1 mL aliquots of plasma and buffy coat were stored in a minus 80°C freezer until further use. Plasma samples from the UK and Germany were collected within 12 months of cfDNA extraction, whereas samples from Italy were archival and collected more than 12 months prior to cfDNA extraction. Samples were collected prior to receipt of TACE (T0), after TACE at the time of first clinic follow-up (T1) and at disease progression (T2). Demographic data (including age and gender), clinical data including aetiology of underlying liver disease, presence of cirrhosis, Child-Turcotte-Pugh score (CTP), Barcelona Cancer Liver Clinic (BCLC) stage, alpha-fetoprotein (AFP) level, presence of portal vein thrombosis and metastases were collected. All patients gave written informed consent in accordance with the Declaration of Helsinki.

### DNA quantification, library preparation and next-generation sequencing

cfDNA was extracted from the plasma using the QIAamp™ Circulating Nucleic Acid Kit (Qiagen, Hilden, Germany) following the manufacturer’s instructions. The concentration of the extracted cfDNA was quantified by DNA fluorometric quantitation using Qubit™ 2.0 system (Life Technologies) and Qubit™ dsDNA HS Assay Kit (Thermo Fisher Scientific). Extracted cfDNA samples were stored at −20 °C until ultra-deep targeted sequencing assay.

The sequencing library was prepared using 1–10 ng of cfDNA. The library quality and size distribution of cfDNA were controlled using a 4200 TapeStation System with the D1000 ScreenTape Analysis assay (Agilent). A custom hybrid capture gene panel comprising 431 regions and 4321 probes (total region size of 277,785 bp), with an estimated coverage of the selected targets of 87.7% were subjected to targeted sequencing using unique molecular identifiers (UMI) technology (Cell3 Target; Nonacus Ltd, Birmingham, United Kingdom). The library was pooled on equimolar amounts and sequenced using 75-bp paired-end run on the Illumina MiSeq™ instrument. The average read depth of 6000x was applied to allow for the detection of rare mutations and accurate estimation of variant allele frequencies.

### Quality control and data pre-processing

Raw paired-end reads were trimmed using UMI-tools; quality control of demultiplexed data was assessed by descriptive statistics and exploratory plots using FastQC (v0.11.9). To produce analysis-ready BAM files, trimmed reads were aligned to the human reference genome GRCh38 with Burrows−Wheeler Aligner (BWA) (v.0.7.17) using default settings. Je’s markdupes was applied to mark and remove PCR duplicate read,s taking UMIs into account. Samtools-flagstat software (v.1.9) and qualimap software (v.2.2.2) were used for a post-alignment quality check^28,29^. Reads with a MAPQ score below 5, PCR duplicates, secondary alignments, supplementary alignments, and unmapped reads were removed prior to further downstream analysis.

The Genome Analysis Toolkit (GATK) and MuTect2 algorithm (Broad Institute, Harvard, US) were employed to call single nucleotide variants (SNVs) and small insertions and deletions (indels). The final candidate variants were manually verified with the integrative genomics viewer (IGV)^30^. Recognised variants listed in human SNP databases (dbSNP, 1000 Genomes) were excluded from analysis unless these variants were also listed in the COSMIC cancer mutation database. We limited our analysis to genetic variants identified in putative genes with a well-established association with HCC, namely TERT promoter (frequency altered in HCC 44%), CTNNB1 (37%), TP53(24%), ARID1A (13%), CDKN2A (9%), AXIN1 (8%), ARID2 (7%), PTEN (7%), ATM (6%), HNF1A (5%), KEAP1 (5%), PIK3CA (4%), RB1(4%), LZTR1 (3%), SF3B1 (3%), APC (1%), JAK1 (1%), and SMARCA4 (1%)^10,11^. Variants were annotated using Funcotator software (Broad Institute, Harvard, USA). Functional annotations in the Ensembl database GRCh38 were identified, and the possible effects of variants were analysed using SnpEff (v5.1)^31^. Somatic variant calling was done as previously described^32^.

### Statistical analysis

As this was a pilot study, no formal power calculation was undertaken. Progression-free survival (PFS) was calculated as the interval of time between the initiation of TACE to the occurrence of either radiological progression or death, whichever occurred first. Overall survival (OS) was determined by measuring the duration of time from the initiation of TACE treatment until death from any cause. To investigate the relationship between prognostic factors and survival rates, both Kaplan-Meier statistics using log-rank test and the Cox regression model were used. Variables with *p* < 0.05 were retained in multivariable analysis. Mann-Whitney test was used to perform nonparametric comparisons, and Student’s T-test was required. All statistical analyses were performed using SPSS version 29 (IBM).

## Supplementary information

Supplementary data

## Data Availability

The sequencing data generated in this study have been submitted to the National Center for Biotechnology Information(NCBI) (NIH, US) and will be publicly available under accession PRJNA1199049 upon publication of this manuscript. Alternatively, all relevant data are available from the authors upon reasonable request.

## Code availability

Codes and pipelines used to analyse sequencing data are available at: https://github.com/Sultan-Imperial/ctDNA-Sequencing-in-HCC-TACE-Response.
