# Supplementary material for: Deep sequencing of circulating tumour DNA as a biomarker of clinical outcome to transarterial chemoembolisation in hepatocellular carcinoma
Source: NPJ Precis Oncol. 2025 Jul 1;9:214. doi: 10.1038/s41698-025-00961-2 (PMC12218101; doi:10.1038/s41698-025-00961-2)
Supplement: Supplementary file 1 — Supplementary data [file 41698_2025_961_MOESM1_ESM.pdf]

Supplementary Table 1. The set of individual mutations status for each patient.

Patient Number	Gene	Variant Classification	Variant Type	dbSNP ID	COSMIC ID	Genome Change
**1**	ARID1A	5'UTR	SNP	rs889462516		g.chr1:26696303T>G
	ATM	3'UTR	SNP			g.chr11:108368056T>C
	ARID2	3'UTR	SNP	rs15852		g.chr12:45907298G>A
	PIK3CA	Intron	DNP		COSV104566301	g.chr3:179204485_179204486CC>AA
	TERT	Intron	SNP	rs1171291378		g.chr5:1282922A>G
	APC	Intron	SNP	rs78919815	COSV57371494	g.chr5:112775613A>T
**2**	ATM	3'UTR	SNP			g.chr11:108366325T>A
	ARID2	Intron	SNP			g.chr12:45776208G>C
	SMARCA4	Intron	SNP			g.chr19:11019395T>C
	SF3B1	Intron	INS	rs749132053	COSV59206637	g.chr2:197400448_197400449insA
	LZTR1	5'UTR	SNP			g.chr22:20982316C>A
	CTNNB1	5'UTR	DEL	rs982253379		g.chr3:41222081delA
	CTNNB1	5'UTR	DNP			g.chr3:41222080_41222081TA>AA
	CTNNB1	5'UTR	INS	rs970901984		g.chr3:41222080_41222081insA
	TERT	5'Flank	SNP	rs1242535815		g.chr5:1295113G>A
	CDKN2A	Missense Mutation	SNP	rs121913385	COSV58682852	g.chr9:21971112G>A
**3**	SMARCA4	Intron	SNP			g.chr19:11025373A>C
	CTNNB1	Missense Mutation	SNP			g.chr3:41233410C>A
**4**	ATM	Intron	DEL			g.chr11:108226129_108226147delCTGTTTCTTTTTTTTTCTT
**5**	SMARCA4	Intron	SNP	rs200916800		g.chr19:10996186A>G
**6**	ATM	3'UTR	SNP			g.chr11:108368079G>A
**7**	ARID1A	5'UTR	DEL	rs1319181968		g.chr1:26696243delG
	ARID1A	Missense Mutation	SNP			g.chr1:26696939C>T
	ARID1A	3'UTR	DEL	rs957140373		g.chr1:26781181delA
	ARID1A	3'UTR	INS	rs1424979331		g.chr1:26781180_26781181insA
	ARID1A	3'UTR	DNP	rs201576837		g.chr1:26781180_26781181CA>AA
	PTEN	3'UTR	SNP	rs935680911		g.chr10:87967851G>T
	TERT	Intron	DEL			g.chr5:1294720delC
**8**	HNF1A	Frame Shift Del	DEL	rs762703502		g.chr12:120994312delG

	TP53	Missense Mutation	SNP	rs148924904	COSV52663142	g.chr17:7675124T>C
	KEAP1	Intron	SNP	rs569540759		g.chr19:10500119G>A
	Unknown	IGR	INS	rs775238241		g.chr2:177227963_177227964insAAAAAAA
	SF3B1	Missense Mutation	SNP	rs559063155	COSV59205318	g.chr2:197402110T>C
	CTNNB1	Intron	SNP	rs1438006044		g.chr3:41237312T>C
**9**	ARID2	Missense Mutation	SNP			g.chr12:45850291T>C
	APC	Intron	SNP	rs78919815	COSV57371494	g.chr5:112775613A>T
**10**	ATM	Intron	SNP	rs765924415		g.chr11:108317331T>C
	CDKN2A	5'UTR	SNP			g.chr9:21995246C>G
**11**	ARID1A	Intron	SNP	rs538175753		g.chr1:26775548T>A
	ATM	Intron	DEL	rs1268617872		g.chr11:108229848_108229850delTTT
	ATM	Silent	SNP	rs551041839		g.chr11:108353824C>G
	AXIN1	Intron	DEL	rs1409165375		g.chr16:292909_292954delTCCCGGATGAGAGAAGGGCACAGTGAGGAGGGCTGTG
	SF3B1	Intron	DEL	rs772961618|rs111354698		g.chr2:197400447delT
	SF3B1	Silent	SNP	rs35493573		g.chr2:197405293T>C
	LZTR1	Silent	SNP	rs145833752	COSV104394373	g.chr22:20992853C>T
	LZTR1	3'UTR	SNP	rs1400712041		g.chr22:20997565C>G
	CTNNB1	5'UTR	SNP	rs9870255		g.chr3:41195090G>C
	PIK3CA	3'UTR	SNP			g.chr3:179237557C>T
	TERT	Silent	SNP	rs1337149388		g.chr5:1255402T>C
	TERT	5'Flank	SNP	rs2853669		g.chr5:1295234A>G
	Unknown	IGR	SNP			g.chr5:112707555G>A
**12**	KEAP1	5'Flank	SNP			g.chr19:10503405C>T
	SF3B1	3'UTR	SNP			g.chr2:197390330T>G
	Unknown	IGR	SNP			g.chr5:112707536G>A
**13**	AXIN1	3'UTR	DEL	rs1237606029|rs61433087		g.chr16:287828delG
	AXIN1	3'UTR	DNP			g.chr16:287827_287828TG>GG
**14**	ARID1A	Intron	SNP			g.chr1:26765502T>A
	PTEN	3'UTR	SNP	rs1165885891		g.chr10:87966104C>T
	SF3B1	Intron	DEL			g.chr2:197419393_197419394delCA
**15**	ARID1A	Missense Mutation	SNP			g.chr1:26780431A>G
	ATM	Intron	DEL	rs1268617872		g.chr11:108229848_108229850delTTT

	SF3B1	Silent	SNP	rs765509812		g.chr2:197398107T>C
	SF3B1	Missense Mutation	SNP	rs754688962	COSV59206172	g.chr2:197402637T>C
	PIK3CA	3'UTR	SNP	rs78993050		g.chr3:179236267T>C
**16**	SF3B1	3'UTR	SNP	rs568846379		g.chr2:197390456G>A
**17**	ATM	Intron	SNP		COSV53720119	g.chr11:108226465A>G
	ARID2	Silent	SNP	rs534769714		g.chr12:45851855C>T
	SMARCA4	Intron	SNP	rs866801196		g.chr19:10961396T>G
	PIK3CA	Intron	SNP			g.chr3:179195993A>G
**18**	ARID1A	Intron	DEL	rs1161815729		g.chr1:26764279_26764281delTTA
	JAK1	Intron	SNP	rs145378558		g.chr1:64869304G>A
	ATM	Intron	DEL			g.chr11:108236535_108236536delAA
**19**	JAK1	3'UTR	SNP	rs186694656		g.chr1:64834085G>A
	JAK1	Intron	SNP	rs310230		g.chr1:64875319G>A
	PTEN	3'UTR	DEL			g.chr10:87966298delG
	AXIN1	Intron	SNP	rs12926617		g.chr16:291664G>A
	AXIN1	Intron	SNP	rs12926629		g.chr16:291690G>A
	AXIN1	Intron	SNP	rs12928131		g.chr16:292771C>T
	AXIN1	Silent	SNP	rs566994263		g.chr16:346333C>T
	KEAP1	Intron	SNP			g.chr19:10503181C>T
	SF3B1	Missense Mutation	SNP	rs754688962	COSV59206172	g.chr2:197402637T>C
	CTNNB1	5'UTR	SNP			g.chr3:41221852T>A
	PIK3CA	Intron	SNP	rs147180420		g.chr3:179197765A>G
	TERT	5'Flank	SNP	rs2735940		g.chr5:1296371A>G
	CDKN2A	Intron	DEL	rs1464533577		g.chr9:21969809delG
**20**	PIK3CA	3'UTR	SNP			g.chr3:179235739C>T
	PDGFRA	Missense Mutation	SNP			g.chr4:54278426T>G
**21**	ARID1A	5'UTR	SNP	rs889462516		g.chr1:26696303T>G
	ARID1A	Missense Mutation	SNP			g.chr1:26775642G>T
	ATM	Silent	SNP	rs772739433		g.chr11:108253979A>G
	SF3B1	Missense Mutation	SNP	rs559063155	COSV59205318	g.chr2:197402110T>C

	TERT	Frame Shift Del	DEL			g.chr5:1294550delG
**22**	ARID1A	Intron	SNP			g.chr1:26766179A>T
	ARID1A	Intron	INS			g.chr1:26771867_26771868insGA
	ARID1A	3'UTR	SNP			g.chr1:26781594G>A
	JAK1	Frame Shift Del	DEL	rs777215086		g.chr1:64860150delG
	ATM	Intron	SNP	rs765924415		g.chr11:108317331T>C
	ATM	Intron	SNP			g.chr11:108336314G>A
	ARID2	Intron	DEL			g.chr12:45893906_45893907delAA
	HNF1A	Intron	DEL			g.chr12:120993492delC
	HNF1A	Intron	INS			g.chr12:120997748_120997749insCCGAGA
	AXIN1	Intron	DEL			g.chr16:290341delC
	KEAP1	Intron	SNP			g.chr19:10489604A>G
	SMARCA4	Missense Mutation	SNP			g.chr19:11007936C>A
	LZTR1	3'UTR	SNP	rs178296		g.chr22:20997892T>C
	TERT	Frame Shift Del	DEL			g.chr5:1253800delC
	APC	Intron	SNP			g.chr5:112828978T>A
	APC	Missense Mutation	SNP	rs769579396		g.chr5:112841202G>A
	APC	Nonsense Mutation	SNP			g.chr5:112843530C>T
	CDKN2A	Intron	SNP	rs757840893		g.chr9:21994124G>A
	CDKN2A	Splice Site	SNP			g.chr9:21994137A>G
	CDKN2A	5'UTR	SNP			g.chr9:21995246C>G
**23**	AXIN1	Intron	DNP			g.chr16:289748_289749CA>AG

Supplementary Table 2. Univariate Cox regression analysis for progression free survival.

	**Progression free survival**		
	Univariate analysis		
	HR	95%CI	p-value
CTNNB1 mutation	1.5	0.2-11.7	0.7
TP53 mutation	**21.5**	1.3-343.7	**0.03**
ARID1A mutation	1.0	0.3-3.6	1.0
VAF	0.9	0.4-1.9	0.7
BCLC	2.0	0.7-5.2	0.2
Presence of PVT	1.0	0.2-4.6	1.0
